# Surgical Operations in Private Practice

**Published:** 1904-11-12

**Authors:** John Aikman


### Hospital Clinics.

### SURGICAL OPERATIONS IN PRIVATE PRACTICE.

By John Aikman, M.D., C.M., Glas.

### II. Compound Fracture and Psoas Abscess.

[illegible]HE earliest attempts in antiseptic surgery were [illegible]ected towards the treatment of compound fracture and psoas abscess. Both of these now fall within the sphere of the general practitioner.

The problem of compound fracture was stated in very simple terms, to induce it to behave as a simple fracture does; the accomplishment required should insure that it behaves even better. It possesses the advantage over psoas abscess that the tissues infected by pyogenic organisms are merely in the stage of inoculation, that they are within reach, that much of the effused blood which has a feeble resisting power, can be removed; and that the tissues are not infected with the tubercle bacillus. We may balance the strain put upon the absorbent system by the reduction of hæmatomata, against the excretion of antiseptic agents absorbed from the wound and the picture is complete. The wound requires protection from septic intrusion, and the limb must have rest for a time.

The responsibility for success rests on the first treatment of the wound which leads down to the fractured bone. There are many methods, but the following may be taken as sufficient. If the bone is projecting scrub it clean with a 5 per cent. phenol solution and a nail brush before returning it to the depths of the wound. If not, introduce a plug of aseptic cotton saturated with the phenol solution into the wound; this should always be the first step. Then draw the skin together over the wound, and if need be, keep it in position with a stitch. Thus we prevent infection of the wound while we cleanse the skin. Rub pine oil over the skin for several inches round the wound, and then ether soap. With a weak solution of phenol work the soap into a lather and use it for shaving the skin and cleansing.

The cleansed surface of the skin should now be covered by a towel wrung out of phenol solution, and the wound opened. There is usually much fat in the fluid which exudes from the wound, and I have seen no harm result from treating it in the same manner as the skin, by pine oil and ether soap; and if there is much ingraining of earth, the mechanical injury caused by the use of a nail-brush which has been sterilised by boiling soon subsides, and septic mischief is avoided. Some remodelling of the wound is an advantage; unimportant structures which are deeply ingrained may be removed, as well as those which are bruised beyond hope of recovery; but great care should be exercised not to remove any bone which is attached to periosteum, which has not been separated from its source of blood supply. Healthy tissues should then be placed in useful position, and the skin inspected. It is a good rule always to pare the edges of the skin; this is less important when the protruding bone causes the wound, than when a contusing external body inflicts it. In the latter case the skin is more apt to be infected by intrusive [illegible]ms, but in most cases the wound made by the surgeon's knife will heal more readily than that produced by the accident. It is often possible to stitch up a wound so treated, provision being made for drainage during the first 48 hours.

Double cyanide gauze with a thin covering of salicylic wool is a good dressing, and there is some advantage in having it not too thick, as the lesser bulk does not interfere with the accurate adaptation of splints. There is little danger in changing the dressings during the first week if the parts are kept in apposition, and there is much to be said in favour of having the wound well on in the healing process by the end of that time. Local anæsthesia is often sufficient for the first dressing, unless the shaft of the bone requires treatment.

Psoas abscess may be taken as the most difficult and complicated form of chronic abscess with wh[illegible] we have to deal. It has, in common with all [illegible] abscesses arising from bone, a tubercular in[illegible] which is often reinforced by other pyogenic organisms. In most chronic abscesses the proliferation of the pus cells raises the tension within the sac sufficiently to cause the liquor puris to exude into the neighbouring tissues, and it carries with it the infecting organisms; but in psoas abscess we have a disorganised vertebral column incapable of supporting the weight of the body, and each time that the spinal support fails, the tension on the sac of the abscess is greatly increased, and the exudation of the liquor puris many times multiplied. Hence it seems that the sac of a psoas abscess is usually slack, and this factor has some bearing on treatment. Some years since Mr. Bernard Roth introduced me to the spinal support described in the writings of Mr. Robert Jones of Liverpool, and manufactured by Messrs. Critchley of that city, and the number of spinal abscesses which have disappeared under its use is a revelation which I can explain in no other way than that the advance of the infection was due to intermittent and powerful pressure from the failing vertebral column, and the recession of it to the altered proportion between the size of the sac and its contents, when the over-distending pressure was removed. With such a support, psoas abscess which has not reached Poupart's ligament seems often to effect its own cure, but so soon as it reaches the groin another factor comes into play, and gravitation secures the pressure needful to its further extension. Direct opening of abscesses which have not appeared in the groin, and all scrapings of the sac, and interference with bone, such as have been successful in the hands of Sir F. Treves, Mr. A. E. Barker, and others are best left to such surgeons whose experience will leave no room for an afterthought on the stairs; but when the sac has reached Poupart's ligament, a simple procedure is open to a careful general practitioner. After cleansing the skin an incision may be made along the inner edge of the sartorius muscle, a little below its origin, and by its guidance to the iliacus. The anterior crural nerve lies between the iliacus and psoas; but a little gentle pressure on the abdomen will indicate where the abscess is nearest the surface, and it may be punctured with a sinus forceps, between the blades of which a sterilised glass tube can be introduced into the cavity of the abscess. Through this tube the contents of the abscess can escape with little chance of infecting the wound. By the same channel some flushing of the cavity may be done, provided that all the appliances used are effectively sterilised, for success depends upon the avoidance of a mixed infection. It is even needful to boil the iodoform used for making the emulsion with which it is good to irrigate the sac, as the iodoform is not free from a suspicion of permitting a growth of bacteria on its surface; though it seems to stimulate the tissues to resist them. The surplus iodoform is then removed, the wound cleansed, and after having been remodelled is stitched up. An aseptic dressing is applied and then a suitable spinal support. In most cases the healing is uninterrupted, and the result satisfactory. I have once seen the sac of a large abscess persist in the thigh, but it disappeared after the aspiration of a remnant of the iodoform emulsion.

